# Frontline healthcare workers experiences and challenges with in-person and remote work during the COVID-19 pandemic: A qualitative study

**DOI:** 10.3389/fpubh.2022.983414

**Published:** 2022-09-20

**Authors:** Holly Sims, Carmen Alvarez, Kimesha Grant, Jessica Walczak, Lisa A. Cooper, Chidinma A. Ibe

**Affiliations:** ^1^Johns Hopkins School of Nursing, Baltimore, MD, United States; ^2^University of Pennsylvania School of Nursing, Philadelphia, PA, United States; ^3^Division of General Internal Medicine, School of Medicine, Johns Hopkins University, Baltimore, MD, United States; ^4^Department of Health, Behavior and Society Johns Hopkins Bloomberg School of Public Health, Baltimore, MD, United States

**Keywords:** frontline healthcare workers, community health centers, COVID-19, stress, organizational support

## Abstract

**Introduction:**

The COVID-19 pandemic created new and exacerbated existing stressors for frontline healthcare workers. Despite being disproportionately affected by COVID-19, little is known about the experiences of frontline healthcare workers serving marginalized populations in community settings.

**Design:**

We used qualitative descriptive methods to understand the experiences of 12 frontline healthcare workers (HCWs) supporting primarily underserved populations in outpatient settings during COVID-19. Interviews were conducted from March to April 2021.

**Methods:**

Interviews were held virtually via Zoom using a semi-structured interview guide. Interviews were audio-recorded, transcribed verbatim, and uploaded into NVivo 12 qualitative data analysis software. The transcripts were dually coded by members of the research team and a thematic analysis was conducted.

**Results:**

Four major themes from the interviews were identified: stressors and burnout, coping strategies, organizational support, and recommendations. HCWs described how the early adjustment period to the pandemic created new challenges as they attempted to navigate changes in the workplace and altered responsibilities at home. HCWs felt largely unsupported by their organizations as they attempted to cope with stressors. Organizational support programs and resources often did not meet frontline workers' needs, and sentiments of unappreciation from leadership contributed to feelings of burnout and frustration as pandemic-related challenges persisted and evolved.

**Conclusion:**

Despite encountering numerous stressors at work and home, resulting from pandemic-related disruptions, frontline HCWs continued to provide care for their clients while navigating emerging challenges. Health organizations should include HCWs in decision-making processes when implementing support systems for workers during times of crisis.

## Introduction

At the onset of the COVID-19 pandemic, frontline healthcare workers (HCWs) were central to the pandemic response and, given their exposure, soon emerged as one of the more affected groups (1). HCWs in the United States not only experienced COVID infections and COVID-related deaths (2), but also, deteriorating mental health (3). Given the sharp uptick in hospitalizations related to COVID-19, the increased stress and burnout among inpatient HCWs has received much attention (4). However, even before the pandemic HCWs—in inpatient and outpatient settings alike—were experiencing high rates of burnout (5). The negative impact of HCW stress and burnout on patient care affirms the importance of learning about the experiences and needs of both inpatient and outpatient HCWs during the pandemic, to better equip healthcare organizations with the tools to provide HCWs with more effective support for support their wellbeing and optimize patient care.

HCWs in outpatient settings and serving marginalized populations warrant greater attention regarding their work experiences during the pandemic, because these workers also represent HCWs who have been most affected by COVID-19. Indeed, researchers have identified that HCWs of color, nurses, and other support staff, as well as those practicing in settings other than hospitals, experienced higher rates of death from COVID-19 (6). Yet, most studies on HCW experiences during COVID have focused on White, inpatient HCWs (3, 7–9). Failing to address the disparate impact of COVID-19 on HCWs has critical implications for members of the workforce and the populations they serve, many of whom were also disproportionately impacted by the pandemic (10).

Furthermore, studies with HCWs indicate that HCWs experienced increased psychological distress for a variety of reasons, including feelings of uncertainty/lack of control, lack of personal protective equipment, inadequate training, and concerns about transmitting the virus to family members (8, 9). Regarding organizational support for frontline workers, HCWs from a medical center reported effective communication with hospital administration keeping them abreast of hospital changes in operations, and initiatives to show support like providing snacks for staff (11). However, these findings reflect issues and support for HCWs onsite, and not for those who worked remotely. Specific to community health centers and organizations serving marginalized populations, healthcare leadership reported challenges with maintaining clinic operations and limited options for supporting their HCWs (12).

The purpose of this study was to understand the experiences of HCWs serving marginalized populations in outpatient settings in Maryland, during COVID, with a particular focus on experiences navigating the pandemic and perceptions of the support they received from their workplace. COVID-19 has laid bare the lack of support, institutional or otherwise, that is available to health care workers who are called on to continue providing care as essential workers, at great risk to themselves. Thus, this study fills an important gap in our understanding of how to anticipate the needs of a workforce that is prepared to meet population health needs during public health crises.

## Methods

### Design

The University Institutional Review Board approved all study procedures. We used qualitative descriptive methodology (13) to explore the personal and work-related stressors experienced by frontline workers during the pandemic. Interviews were conducted by trained research nurses with qualitative research experience.

### Recruitment and data collection

We recruited a convenience sample of HCWs using email announcements. We collaborated with healthcare leadership from collaborating healthcare organizations in Maryland to distribute the study announcement to their staff. The study announcement included a hyperlink for an online REDCap survey that included a consent form, eligibility screening, a demographic questionnaire, and the option to leave contact information if interested in participating in the interviews. The study team also made announcements at morning huddles to invite participants to apply. Eligibility criteria consisted of adults: (i) over 18 years of age; (ii) employment for at least a year at healthcare or health service organization serving primarily marginalized populations, (iii) working as a nurse, medical assistant, social worker, or community health worker, (iv) English or Spanish speaking. Participants who indicated interest in being interviewed were contacted by email to schedule the interviews.

### Data collection

Of the nineteen people who indicated interest in participating in the interviews, twelve individual interviews were conducted between March and April 2021. Informed consent was obtained electronically prior to the interviews. The interviewers used a semi-structured interview guide containing open-ended questions to facilitate the discussion (Table 1). The participants were asked to describe stressors experienced during the pandemic, changes in the work environment and delivery of patient care, and the degree to which they felt supported by their organizations as they attempted to cope with challenges at work and at home. Participants were prompted to expand on any comments that were particularly insightful or relevant to the study's aims. All interviews were conducted *via* ZOOM and audio-recorded, with the participants' consent. On average, the interviews were 1 h in duration. The recordings were transcribed verbatim and de-identified.

**Table 1:** Semi-structured interview guide.

**Question**	**Examples of probes**
**Focus area: work-life**	
Tell us about your current position.	What are your responsibilities?
	Describe your work environment.
	How would you describe the patients for whom you provide care?
Before COVID-19, what were the things that you enjoyed about work?	What did you not enjoy?
Since COVID-19, what things have changed with your job?	How have your responsibilities changed?
	What things have you had to learn to do your job?
	How have you felt about these changes?
	What types of support or help have you received to deal with these changes?
	How has COVID-19 affected your ability to care for your patients?
Regarding the patients you serve, since COVID-19, what have been their greatest needs?	How if at all are you able to help them with these challenges?
**Focus area: home-life**	
Before COVID-19, how would you describe your home life?	What are your responsibilities within the home?
	What did you enjoy at home?
	What if anything did you find stressful at home?
How has your home life changed since COVID-19?	How would you describe the stress level in the home?
	How have you been able to manage your child(ren's) needs?
	How have other adults in the home been able to contribute to the household?
**Focus area: sources of support**	
What types of support do you need right now?	From where or from whom do you think you should receive support or help?
	How should your job be supporting you right now?
	How should the government be supporting you?
	How should family be supporting you?

### Thematic analysis

The study team conducted a thematic analysis of the interview transcripts to identify and analyze patterns in the data and draw inferences from the emerging themes. Specifically, we employed a systematic, phased approach to the analysis that was consistent with guidelines proposed by Braun and Clarke (14). The interview transcripts were first read independently by all members of the research team, while noting initial ideas and potential codes reflective of the data. The study team then met weekly to facilitate the inductive data analysis, beginning with a collective discussion of the themes and codes identified independently. An initial codebook was generated collectively, which included detailed descriptions of the codes and supporting examples from the data. A dual coding process was then conducted using NVivo12 qualitative data analysis software; interview texts were independently coded by two members of the research team, after which the coded data was merged using NVivo's coding comparison feature to assess for inter-rater reliability. Discrepancies were discussed as a group, and the codebook was modified to remove redundancies and clarify codes.

Throughout the coding process, the study team independently identified themes that emerged from the data. The study team discussed thematic elements within the interview transcripts throughout the coding process, after which the most significant themes that emerged from the data were collectively identified and defined. Relevant codes and examples from the data were gathered to support each theme and refine the naming or definition of the theme when necessary, ensuring that the themes reflected patterns and stories present across the entire data set. A final report of the thematic analysis was then produced, containing extracts from the texts, and summarizing the ways in which our analysis related to the initial study aims.

### Rigor

Several strategies were used to ensure trustworthiness and rigor throughout all aspects of study implementation (15). A semi-structured interview guide was developed to ensure that all data of interest was collected during the interviews through a series of open-ended probes. The study team members reviewed and discussed the interview guide before data collection to ensure that all members were familiar with the probes. Prior to conducting participant interviews, the study team reviewed best practices for qualitative data collection and conducted practice interviews to gain familiarity with qualitative interviewing techniques. The interview audio recordings were transcribed verbatim using a professional transcription service, which was further reviewed for accuracy by the study team by cross-checking sample recordings with the corresponding transcribed text.

We developed a codebook to standardize the coding process; the codebook comprised detailed descriptions for the application of each code as well as sample interview text to serve as a reference. Rigor was established during the data analysis process by assigning two study team members to independently code interview texts, after which comparison of coding was conducted using NVivo12 software. Any remaining discrepancies were resolved collectively during weekly study team meetings held throughout the data analysis process.

## Results

Participants (*N* = 12) were mostly female (*n* = 10) and ranged in age from 32 to 62 years old (average 45 years). Participants were nurses (*n* = 4), community health workers (*n* = 5), social workers (*n* = 2), and a medical assistant. They self-identified as Non-Hispanic White (*n* = 2), Non-Hispanic Black/African American (*n* = 4), and Hispanic (n = 6) (Table 2). Nine participants were employed by community health organizations, 2 worked in inpatient settings, and 1 worked in an emergency department. All participants provided services to lower-income or marginalized populations (e.g., undocumented, homeless).

**Table 2:** Participants' job titles and work setting.

Participant 1	Community Health Worker, Healthcare Service Organization
Participant 2	Social Worker, Outpatient Pediatric Clinic
Participant 3	Registered Nurse, Intrapartum
Participant 4	Medical Assistant, Community Health Clinic
Participant 5	Community Health Worker, Healthcare Service Organization
Participant 6	Licensed Practical Nurse, Community Health Clinic
Participant 7	Registered Nurse, Inpatient Perioperative Care
Participant 8	Community Health Worker, Community Health Clinic
Participant 9	Community Health Worker, Healthcare Organization
Participant 10	Social Worker, Outpatient HIV Clinic
Participant 11	Registered Nurse, Emergency Room
Participant 12	Community Health Worker, Healthcare Organization

To summarize, the participants described numerous challenges they faced as they attempted to navigate the changes brought by the pandemic. They relayed feelings of stress and burnout resulting from altered or increased responsibilities at work and at home. HCWs felt that support from organizational leadership was inadequate to address many needs such as childcare assistance, protection from exposure, and resources for mental health. They described feeling underappreciated by their leadership, who often failed to acknowledge or address the increased workload and risks for exposure to COVID-19. Stressors and altered responsibilities at home, such as managing children's virtual schooling and fear of exposing family members to the virus, further contributed to feelings of burnout. To cope with pandemic-related stressors, many frontline workers sought their own forms of self-care including taking time off, pursuing hobbies, and seeking mental health services or other sources of emotional support. Our findings are organized under four major themes: stressors and burnout; coping strategies; organizational support; and recommendations.

### Stressors and burnout

#### Work-related stressors

At work, participants reported an ever-changing environment that upended work operations and how they delivered patient care. Some participants reported receiving little information on the rationale behind the changes and felt excluded from decision-making. Participants expressed frustration with the lack of consistent information, constant shifts in protocols, and mixed messages from organizational leadership:

“Just that literally from a day-to-day basis, you can show up, and things are being done different than they were the day before when you were there. From shift-to-shift, you're getting different information. Then now also, every day you're being blasted with new information, long memos about new procedures about things.” [Registered Nurse, 3].

“I think for me, my biggest issue has been the response from the hospital. At the beginning it was like no masks are needed, then yes, masks are needed. They now act like it was always that the masks were needed, but no, that was not the original message. I feel like although I can recognize that information evolves, I don't feel like it has evolved as much as the mixed messages.” [Social Worker, 2].

HCWs who continued with patient-facing services expressed multiple work-related changes. These changes included new responsibilities: for example, HCWs who used to do community outreach for HIV prevention changed to providing COVID-19 prevention outreach. One community health worker who used to offer health promotion services and addressed patients' health-related social needs (e.g., housing and food insecurity), had her role changed to screening patients coming into the clinic. The additional task of screening patients for COVID-19 symptoms was frequently cited by our study participants. Workers found screening patients to be extremely stressful for a variety of reasons. Chief among them were patients not complying with physical distancing or wearing a mask and having to work with crowds of people in an enclosed space. Moreover, screening patients contributed to longer working hours. For example, the medical assistant explained that she had to arrive at work 45 minutes early to screen incoming patients and that doing so was a new responsibility in addition to her usual tasks. She expressed her feelings about the new workload: “*If anything, I*'*ve felt like I*'*ve had more added duties and with no more compensation to go with it. You*'*re putting my life in danger, and then I*'*m getting more duties.”* This sentiment dovetails with several participants' reports of taking on longer working hours due to personnel shortages and additional tasks outside of their typical scope of work, at the expense of their physical and mental well-being.

Fear of contracting and transmitting the virus to family members was another concern among patient-facing HCWs. As one social worker shared: “*I used to keep my clothes separate from my husband*, '*cause I thought, ‘I'm a virus walking around.' We didn't know a lot, so I used to worry about bringing the virus home.”* Other participants shared similar concerns and were particularly worried about those who were more medically vulnerable within their homes, such as their elderly parents, or children with asthma. Although personal protective equipment (PPE) was provided, HCWs remained preoccupied with fears of spreading the virus to loved ones:

“Even with the PPE, I still felt like I wasn't being protected, and it was always that fear of am I gonna get this from coming in contact with people? People would come. They would come closer. Then they wouldn't respect the six feet boundary.” [Community Health Worker, 8].

Having to re-use PPE due to scarcity of supplies also contributed to HCWs' concerns about the integrity and effectiveness of PPE for preventing transmission of COVID-19. One participant shared an extreme situation: “*...N95, we were using them for 30 days then getting them sterilized an additional two times for a total of 90 days use*..” (Registered Nurse, 11). This nurse went on to explain that if a worker tested positive from COVID and it was determined that it was from incorrect use of PPE or breach in PPE, then they were not entitled to be paid for their time away.

Working from home also had its share of challenges. Healthcare workers who transitioned to full telework now had to learn new systems that created challenges performing what were once routine work activities. One participant reported that due to telework, communication and work efficiency decreased, which affected the timeliness with which she could attend to client needs:

“…you couldn't go onto social services office. You had to either try to leave a message, or get online and file applications online and then take forever to get a response or to get action. Things just slowed down for everybody. You still try to do what you can do, but some things it's just outta my control.” [Community Health Worker, 5].

Other community health workers and social workers shared similar frustrations about not being able to help their clients more efficiently. For example, filling paperwork to help a client obtain a service now required the CHW to download the form, print it, and mail it to the client if they did not have a computer to complete the process themselves.

The transition to remote work often removed the work-home boundaries. Some participants recalled feeling as if they were always at work, with the attendant difficulty of mentally escaping work when it existed in the same place as home:

“I think mid-COVID, it was stressful because, like I said, working. My wife's working. I'm in a meeting. She's in a meeting. We live in a townhouse, so she had to make a space downstairs. At the beginning of COVID, I was in our room. I was getting depressed because I've been in a room all day. Working in your room all day, then going to sleep in your room. I never was out of that box of my room.” [Community Health Worker, 1].

Work also encroached on personal equipment, where personal phones and laptops were now being used for work, rather than solely for personal use.

#### Personal-related stressors

Many work-related stressors also contributed to challenges at home—both for those who worked onsite and for those who worked remotely. Personnel shortages contributed to some having to work more hours, which subsequently impacted their ability to function at home:

Sometimes, I'm on call right after a shift. I am doing a 12-hour shift and then an 8-hour shift on top of that. I'm doing 20 hours, and by the time that I come home, it may be in the morning. I am barely coherent to really talk to the kids or make breakfast or make coffee, but I'm still trying to manage those things. [Registered Nurse, 7].

Participants working remotely with school-aged children at home also reported the constant tension of having to be attentive to both their professional duties and their children's needs. One community health worker shared that her child has issues with attention and reported consistently having to monitor him while also trying to do her work. Some participants reported feeling as though they were always working and not spending enough time with their children. They also described how their children started expressing their frustrations about their parents' increased work hours:

My son was at school in the school at home [virtual learning]. I was working all day. He was very resentful for a while about me working that much…He cried, and he said, “Mommy, I know that you're doing a good thing, but I need you.” That was very, very hard. I was there, then I wasn't really. [Community Health Worker, 12].

Participants working from home also reported tensions with spouses, in part because of differing perspectives on the work-life balance when working remotely. Fueling this tension was the lack of familial support due to COVID-19 restrictions. While some participants reported having family members who were willing to help them with their children, maintaining COVID-19 precautions and concerns about transmitting the virus to others deterred them from pursuing such assistance.

At the other end of the spectrum, isolation was a challenge for those without children or other family members at home. Several participants reported difficulty adapting to not seeing their loved ones and engaging in their usual pre-pandemic social activities. Virtual social events were cited as a valuable reprieve by some participants, but others did not find them helpful, particularly as they were on frequent Zoom sessions for work.

We identified a discernable difference in the level of stress expressed by the community health workers and the medical assistant, compared to the nurses and social workers. Notably, the community health workers and medical assistant did not share as many options or resources as the other professionals for managing their stressors. For example, the nurses and social workers discussed being able to reduce work hours, hire assistance for home responsibilities, and have groceries delivered. In contrast, supports to mitigate the impact of COVID-related stressors were not mentioned as frequently by the community health workers and the medical assistant.

#### Burnout

In addition to reporting work and home-related stressors, participants also described symptoms consistent with burnout. Several participants explicitly mentioned feelings of frustration, exhaustion, and low motivation:

I know that I'm not at home as much, and I feel guilty and slightly resenting work that things have gotten so bad, but it's not their fault. I wish that I could be more at home, and I think that I've broken down a couple of times in tears because I just wanna be home. [Registered Nurse, 7].

Other participants reported finding it harder and harder to get out of bed to go work, constantly feeling tired and exhausted. Some who worked at home shared similar sentiments: “*I just feel so burned out. I feel so tired. I don't know. I want it to end. I'm tired of working from home. This needs to stop*.” [Community Health Worker, 1]. In addition to these symptoms of burnout, several HCW shared that they were dealing with depression and anxiety pre-pandemic, and their symptoms worsened with the multiple stressors of the pandemic.

### Coping strategies

In discussing their many challenges, participants' strategies for coping emerged. These strategies included keeping a positive attitude and being adaptable:

For the most part, I say that one of my superpowers is my ability to adapt. I'm very flexible. I can quickly compose myself and be like, ‘Okay. Teach me this new way of doing things, so that we can carry on with our day. [Registered Nurse, 3].

Other participants mentioned gardening, exercising, and taking frequent breaks from working on the computer. One participant shared that she started utilizing her acquired vacation and sick days more to have more respite time: “*I have a lot of sick time and vacation and personal days. I really try to incorporate that monthly now. It*'*s a decided thing that I found I need as part of my coping mechanism.”* [Community Health Worker, 8]. Others also reported using paid time-off also for the purpose of having a “mental break,” and reducing their hours to change their status from full-time to part-time employment.

Some participants endorsed the desire for mental health resources such as therapy; however, accessing these services was a continuous challenge. For example, one participant stated that they tried to get an appointment for therapy, but appointment times were only offered during standard business hours, which conflicted with their work schedule. Overall, most participants reported engaging in positive, healthy coping mechanisms. However, one participant mentioned that their alcohol consumption had increased:

I'm in therapy, so—and I have been in therapy even before COVID. I was doing that once a week, and I take medication for anxiety, for depression. I think that I was lucky to have that in hand, anyways, and some drinking, too. That increased. [Community Health Worker, 12].

### Organizational support

HCWs reported a variety of resources when asked about how they were being supported during the pandemic. For some who transitioned to remote work, their organization provided equipment such as printers and PPE for their outreach activities. One organization provided an end-of-year bonus; as part of an appreciation week, another provided gift cards and lunches. Because of the pandemic, another organization allowed employees to take time off, with the caveat that it was without pay. Additionally, some organizations convened virtually held group meetings to foster social support, while others maintained existing services like employee assistance programs that offered as-needed counseling.

Some participants reported availing themselves of supplemental profession-based support through virtual workgroups, which were convened by the organizing bodies of their profession to foster sharing and support around common issues. Activities included yoga, meditation, and/or opportunities to discuss issues with their peers. As one participant stated:

I was fortunate enough that we used to do Zoom calls among the CHWs, and we did different webinars where we did meditation, and we did some relaxation, yoga, like chair yoga and different things, and that did help. It did, and then we would have this thing where we just do check-ins, too, where we got a chance to just rant [laughter] for 15, 20 minutes, and that was good (Community Health Workers, 8).

Other than these formal forms of support, participants expressed strong appreciation for the informal and emotional support received from coworkers and sometimes-immediate supervisors more. One nurse shared: “*The most support and help just comes from us in the cubbies here, and it*'*s the most meaningful kind of, I think, support because you know it*'*s honest when you get it*. [Laughter]” Another mentioned that her manager and leadership were “outstanding” and “absolutely phenomenal” in part due to their “open-door policy” that allowed HCWs to come and share their thoughts about necessary changes. They also provided weekly opportunities for staff to share their concerns:

On Thursdays, we do in-service. Sometimes, we do social distance in-service. There was one at the in-service that we gathered six feet apart in the OR hallways, and we spoke of the things that we were concerned about, what things we felt like we've been doing good. Were we struggling with anything? Do we see any changes that need to happen? Then we danced… [Registered Nurse, 7].

Another participant also reported feeling very supported by her supervisor. This was a unique case where she was able to call her supervisor even after hours.

### Recommendations

When asked how their organizations could support them during the pandemic, participants' suggested healthcare leadership demonstrate more empathy and appreciation for the workforce, and to be more inclusive of frontline healthcare workers in their decision-making. A lack of appreciation from leadership was the most common criticism voiced by participants. They felt that organizational leadership did not acknowledge how frontline workers managed the increased workload and challenges. HCWs expressed wanting to receive more gratitude from leadership, a sentiment captured by the Medical Assistant: “*If they would let us know, “Hey, you*'*re doing a great job… I know you have people you go home to too, but we just appreciate you for hanging in there.”* Other participants also shared that they would have appreciated more communication between leadership and frontline workers so that leadership could recognize and acknowledge the day-to-day challenges that frontline workers confronted. One participant wanted healthcare leadership to go beyond verbal acknowledgment, “*having your employer…not just call you heroes, but treat you like one.”* (Registered Nurse, 11). Actions that would demonstrate greater appreciation included better protections against exposure including appropriate PPE, more stringent screening practices, providing a clean environment, and pay while recovering from COVID-19. Some participants also suggested additional financial support for managing additional responsibilities and expenses incurred by the pandemic:

“...monetary support like my phone. I mentioned it to leadership before, and they're like, “Well, it's a privilege to be home then,” and I'm like, “Just 'cause you have a job doesn't mean I'm financially well off.” I feel like sometimes that people think that, “Oh, you have a job, so you don't need food, or you don't energy assistance, or you don't need rental assistance, or something like that.” I feel as far as my phone, I pay my phone each month, and a majority of my calls, and what I do is for work. [Community Health Worker, 1].

Another community health worker suggested that much of the lack of appreciation from healthcare leadership was because of the nature of their work. He explained that community health workers and other workers were like “second-class citizens” because they did not provide inpatient clinical care. He believed that much of the healthcare leadership only valued the work of doctors, nurses, and other first responders, and overlooked the other workers keeping the hospitals clean, serving food, and the community health workers “*who are out there tryin' to fill in gaps for those people who can*'*t get to the hospital…who are scared to go to the hospital*.”

The other main recommendation emerging from the participants was to include frontline workers in the decision-making process when creating systems and protocols to support workers during times of crisis. Many participants reported frustrations with rules and protocols, in part because they did not understand how leadership was making decisions. For example, some participants expressed resentment over having to be onsite while others were allowed to work remotely. There were also frustrations about decisions for discontinuing remote work even after it was clear that being onsite was not essential for completing many responsibilities—especially for those who provided other support and outreach services. One participant commented:

“It would've been nice if they would've had us at the table. If they really are about all the things that they say they are, they would've had us at the table and heard what our concerns were and try to have us there talking about it and try to figure out a way to do what we gotta do here.” [Social Worker, 10].

## Discussion

Our study is an important contribution to the growing evidence exploring how COVID-19 impacted frontline workers during the COVID-19 pandemic, particularly those working in outpatient settings with populations most adversely affected by negative social determinants of health and grappling with emergent and chronic conditions. Unique to our study was the inclusion of non-medical frontline workers, inquiry about both personal and work-related challenges, and organizational support for their challenges. Even though working remotely and onsite presented different challenges, both settings created multiple stressors that contributed to exacerbating psychosocial distress among the workers. Our findings suggest that although organizations provided some technical support for addressing COVID-19 related challenges, healthcare workers—practicing remotely or onsite—still felt underappreciated and burned out. Nonetheless, they continued to serve their roles and mainly recommended that organizational leadership demonstrate more appreciation and practice more inclusivity in their decision-making.

Our findings on work-related stressors align with other studies that examined stressors during the COVID-19 pandemic among clinical and non-clinical staff (16, 17). Challenges with scarcity of PPE, fear of transmitting COVID-19 to loved ones, and feeling overworked and burned out were also reported by other healthcare workers from inpatient settings (11, 16). In addition, quantitative data that included non-clinical staff indicated that stress, symptoms of burnout, and not feeling valued were experienced differentially among the workforce, with ethnic minoritized groups reporting higher levels stress, burnout, and feeling undervalued (16). Even with our sample size, we also noted that the community health workers and medical assistant—regardless of work-setting (onsite or remote)—expressed more feelings of stress compared to the other workers (nurses and social workers). As stated by one of the community health workers, having employment in and of itself does not mean that one always has the resources to address their challenges. Therefore, when considering resources for the spectrum of healthcare workers, healthcare leaders ought to be intentional about the provision of comprehensive resources, particularly for workers on the lower end of the pay spectrum. A tailored approach to supporting frontline workers during the pandemic or other times of crisis may be appropriate, with the understanding that blanket initiatives or support services may not equally benefit all workers.

Our participants provided practical suggestions for healthcare leaders to consider to effectively support healthcare workers and demonstrate their appreciation for frontline HCWs' efforts. Despite the plethora of challenges healthcare workers experienced during the COVID-19 pandemic, few studies have presented potential solutions for leaders of healthcare organizations. Participants presented actionable feedback, such as offering words of appreciation, demonstrating care by providing optimal PPE, and creating opportunities for more open communication between leadership and frontline workers by including frontline workers in decision-making processes. Inpatient healthcare workers identified organizational transparency, as well as actions for stress mitigation, as practices that relay feelings of support and appreciation (11). These suggestions, alongside those made by our participants, align with expert suggestions on reducing burnout in healthcare workers (18, 19). However, interviews with healthcare leadership (12) about how they were supporting frontline workers suggest that they were not aware of these stress reduction/resilience promotion practices. Also, the uncertainty and constant changes brought by the pandemic may have stifled organizational leadership ability to adapt and accommodate the needs of their workers, especially when simultaneously attempting to maintain operational functions and patient care services. Including HCWs in the decision-making process and asking for their feedback on the success of organizational efforts may have augmented the impact of the leadership's initiatives in supporting workers.

The comments we heard from our participants suggest that there may be a differential risk for burnout among healthcare workers, with community health workers being at relatively increased risk. This finding aligns with a recent commentary from the President of the National Community Health Worker Association, who cites the unique intersections of racism, classism, and sexism experienced by a frequently overlooked workforce predominated by low-income ethnic minority women as fundamental to understanding their pandemic experiences (20). These antecedent factors, combined with unstable funding and persistent challenges integrating community health workers into healthcare settings (21), likely paved the way for our community health worker participants to experience the pandemic differently than their counterparts. It suggests a need to galvanize support for policies and practices that promote community health workers' overall well-being and is a health equity issue in its own right (22). The lessons learned from the stressors and challenges described by the HCWs in this study demonstrate a need to account for how working during times of crisis alters HCWs' personal circumstances and ability to cope with stress. The participants had various recommendations for leadership on how they may have better supported their needs, noting that although challenges were faced at both work and at home, the focus on work-related support neglects other areas of their life that require attention. Healthcare leadership would benefit in the future from acknowledging the relationship between work and personal stressors, and how supporting multiple facets of HCWs' lives will retain them and improve their ability to provide quality patient care. This recommendation is consistent with the quintuple aim for health care improvement, which includes reducing health care cost, improving patient outcomes, improving patient experience, and achieving equity (23). These aims are unachievable without a functional and thriving healthcare workforce.

### Limitations

Our study had several limitations. The fluidity of the pandemic creates challenges in the ability to generalize results, given that the participant responses reflect only the time at which the interviews were conducted. Most of these interviews were conducted prior to widespread vaccine availability; therefore, the impact of community vaccination may have altered the stressors or other sentiments voiced by participants, particularly in relation to fear of virus exposure or social isolation. Furthermore, our sample included participants mainly women from one state. As such, the results may not be representative of the views or experiences of all frontline healthcare workers.

## Conclusion

Existing literature has primarily focused on the impact of COVID-19 on acute care workers. However, HCWs in community and outpatient settings were also required to adapt to the challenges of the pandemic and continue providing critical services to meet the needs of the underserved populations for whom they cared. These frontline HCWs experienced COVID-related burnout and numerous stressors at work and at home, yet organizational leadership did not recognize many of these challenges. Consequently, the types of support available from healthcare leadership did not address the diverse range of stressors. This study highlights the need to incorporate the voices and opinions of HCWs when healthcare organizations design and implement programs to support worker wellbeing as the COVID-19 situation evolves and during future times of crisis. Community and outpatient HCWs were foundational to the initial COVID-19 response and will continue to be instrumental in meeting the rising needs in the community that the pandemic has created and reinforced.

## Data availability statement

The raw data supporting the conclusions of this article will be made available by the authors, without undue reservation.

## Ethics statement

The studies involving human participants were reviewed and approved by Johns Hopkins School of Medicine Institutional Review Board. Written informed consent for participation was not required for this study in accordance with the national legislation and the institutional requirements.

## Author contributions

Conceptualization of study and funding acquisition: CA and LC. Methodology: CA. Formal analysis, data curation, writing—original draft preparation, and project administration: CA, HS, KG, and JW. Writing—review and editing: LC and CI. All authors have read and agreed to the published version of the manuscript.

## Funding

This research was funded by the Johns Hopkins Alliance for a Healthier World and the Urban Health Institute.

## Conflict of interest

The authors declare that the research was conducted in the absence of any commercial or financial relationships that could be construed as a potential conflict of interest.

## Publisher's note

All claims expressed in this article are solely those of the authors and do not necessarily represent those of their affiliated organizations, or those of the publisher, the editors and the reviewers. Any product that may be evaluated in this article, or claim that may be made by its manufacturer, is not guaranteed or endorsed by the publisher.
